# Influence of urban morphology on COVID-19 transmission in Saudi Arabia: lessons for the post-COVID era

**DOI:** 10.3389/fpubh.2026.1825759

**Published:** 2026-06-15

**Authors:** Arwa Alumran, Shayma Alumran, Mashary Al-Naim, Ahmed Alkhoudiri, Deema Al Shawan

**Affiliations:** 1 College of Public Health, Imam Abdulrahman Bin Faisal University, Dammam, Saudi Arabia; 2 College of Design, Imam Abdulrahman Bin Faisal University, Dammam, Saudi Arabia; 3 College of Architecture and Planning, Imam Abdulrahman Bin Faisal University, Dammam, Saudi Arabia

**Keywords:** COVID-19, density, infection, pandemic, public health, urban fabric, urban mobility

## Abstract

**Background:**

Urban environments may influence COVID-19 transmission.

**Objective:**

To examine how urban form, population size, and density relate to COVID-19 spread in Saudi Arabian cities.

**Methods:**

A cross-sectional study of 18 major cities categorized as centralized, linear, or grid-patterned. Data on confirmed cases, population, area, and density were analyzed using descriptive statistics and correlation analyses.

**Results:**

Total COVID-19 cases strongly correlated with city population size (*r* = 0.942, *p* < 0.001) and moderately with cases per density (*r* = 0.506, *p* = 0.032). Population density alone was not significant. Centralized cities exhibited higher infection rates than linear or grid-patterned cities.

**Conclusion:**

Population size drives COVID-19 spread, while urban form and connectivity shape local transmission dynamics. Urban planning and targeted interventions in centralized areas can support pandemic mitigation.

## Introduction

As stated by the World Health Organization (1), the coronavirus disease (COVID-19) is a highly contagious communicable disease brought on by the SARS-CoV-2 virus, first observed on December 31, 2019, in Wuhan, China. Typically, the illness begins with mild symptoms of fever, dry cough, and fatigue, while serious illnesses can damage the respiratory tract and may bring about demise. By the end of the first quarter of 2020, COVID-19 was proclaimed a worldwide outbreak, and by the end of 2021, more than 280 million cases and almost six million fatalities had been documented globally (1). In Saudi Arabia, there were 553,912 cases documented by 31 December, 2021, and a full vaccination level of 66.39% among every 100 individuals. Early community health measures, introduced from March 2020, drastically lowered regional counts of instances and diluted the possibility of expansive scale outbreaks (2). In spite of this, rates of infection differed among major cities, and thus there was a need for analysis of the role of urban form in COVID-19 transmission.

Saudi Arabia implemented a comprehensive and proactive public health response during the COVID-19 pandemic, which included early travel restrictions, suspension of religious gatherings, curfews, remote education policies, digital health surveillance systems, and extensive vaccination campaigns. These interventions were coordinated through national disaster management and public health frameworks led by the Ministry of Health and other governmental sectors (2). The Saudi experience demonstrated the importance of rapid policy implementation, intersectoral collaboration, and localized preventive strategies in mitigating disease transmission across cities with varying demographic and urban characteristics. In addition, the pandemic highlighted how urban mobility, population concentration, and city connectivity can influence the effectiveness of public health interventions. Understanding these interactions is essential for improving future pandemic preparedness, strengthening urban resilience, and informing evidence-based urban planning and public health policies in Saudi Arabia and similar rapidly urbanizing settings (3).

Urban form can be described in terms of structural and transportation attributes of a city (3). Cities in Saudi tend to be characterized into three forms of urban form, namely, linear, grid, and radial. Historically important cities, for instance, tend to have central urban fabrics, and new development usually takes place around old core centers. Linear cities, for example, Jeddah and Najran, usually occur on coastlines or mountain ridges, while grid-patterned cities, e.g., Dammam and Tabouk, reflect more modern forms of urban planning. It will be observed, given variance in population and urban growth, that pedestrian usage continues to be concentrated in culturally, religious, and economic centers of importance. Private transport usage of public transit continues to prevail in these cities.

COVID-19 has also triggered a fast growth of published scientific literature. By December 2021, a search of COVID-19 in scientific databases produced 100,837 papers, and most of these were in medicine, immunology, and microbiology. Research on the relationship between urban environment and disease transmission was limited before this pandemic (4). During the past few decades, initiatives toward increasing the density of cities have attempted increasing efficiency and reducing greenhouse gas emissions (5). Now, more than 55% of the world’s population lives in cities, and this is estimated at increasing to 66% of the global population by 2050 (6). Therefore, urban density is a significant variable in describing the characteristics of cities pertinent to transmission of disease.

Some studies have found a positive correlation between density and infection rates (3, 7–12). Dense locations provide favorable conditions for transmission of pathogens and, as such, promote more horizontal growth of the city for epidemic risk prevention (13, 14). Moreover, Carozzi (15) showed density impacts outbreak time, and those cities denser in population tended to have earlier outbreak time. Other studies, however, showed no significant correlation between COVID-19 transmission rates and density (7, 16–18), and, accordingly, density may not solely be responsible for variation of number of cases variation. Density may instead interact through other geographic and demographic factors, such as levels of urbanization and aged population percentages, in determining infection rates (19). Little work on the impact of city form and fabric of the city in the transmission of disease is still carried out.

The record-breaking scale of the COVID-19 pandemic has spurred intensive scientific investigation of its transmission dynamics, its environment and socioeconomic consequences, and recovery and adaptation strategies. Although various studies have estimated COVID-19 transmission in Saudi Arabia, no study has comparatively assessed urban fabric of cities regarding case numbers explicitly. Therefore, this study attempts to explore COVID-19 infection rates considering urban fabric, city density, and mobility patterns for Saudi Arabia through the synthesis of literature review, official sources of data, and statistical analysis. Due to the large difference in the number of cases between Saudi cities, it is critical to understand the role of urban attributes. It is the first objective of this investigation to find if there is an effect of the cities’ urban attributes on the transmission of the COVID-19 pandemic.

Although the acute phase of the COVID-19 pandemic has subsided, understanding the spatial and urban determinants of infectious disease transmission remains highly relevant for future pandemic preparedness. The COVID-19 pandemic highlighted vulnerabilities in urban systems, prompting renewed attention to how city design, mobility patterns, and population distribution influence disease spread. Lessons from COVID-19 can inform resilient urban planning strategies that mitigate risks during future infectious disease outbreaks. This study provides one of the first comparative analyses examining the relationship between urban morphology and COVID-19 transmission across major Saudi cities. By examining variations in centralized, linear, and grid-patterned urban structures, the study contributes to understanding how urban design influences infectious disease transmission and offers insights for post-pandemic urban resilience planning in rapidly urbanizing regions.

## Methodology

This study employed a cross-sectional design to examine the distribution of COVID-19 cases across major cities in Saudi Arabia from the start of the pandemic up to November 2021. Eighteen cities were purposively selected to ensure representation across municipal jurisdictions and geographic regions of the Kingdom. These cities included Jeddah, Makkah, Riyadh, Jubail, Al Madinah, Dammam, Abha, Tabuk, Buraidah, Hail, Arar, Jazan, Najran, Al Baha, Sakaka, Taif, Al Hofuf, and Hafar Al Batin. Collectively, the selected cities represent diverse urban morphologies, including centralized, linear, and grid patterns, as well as considerable variation in population size and density.

**Figure 1:**
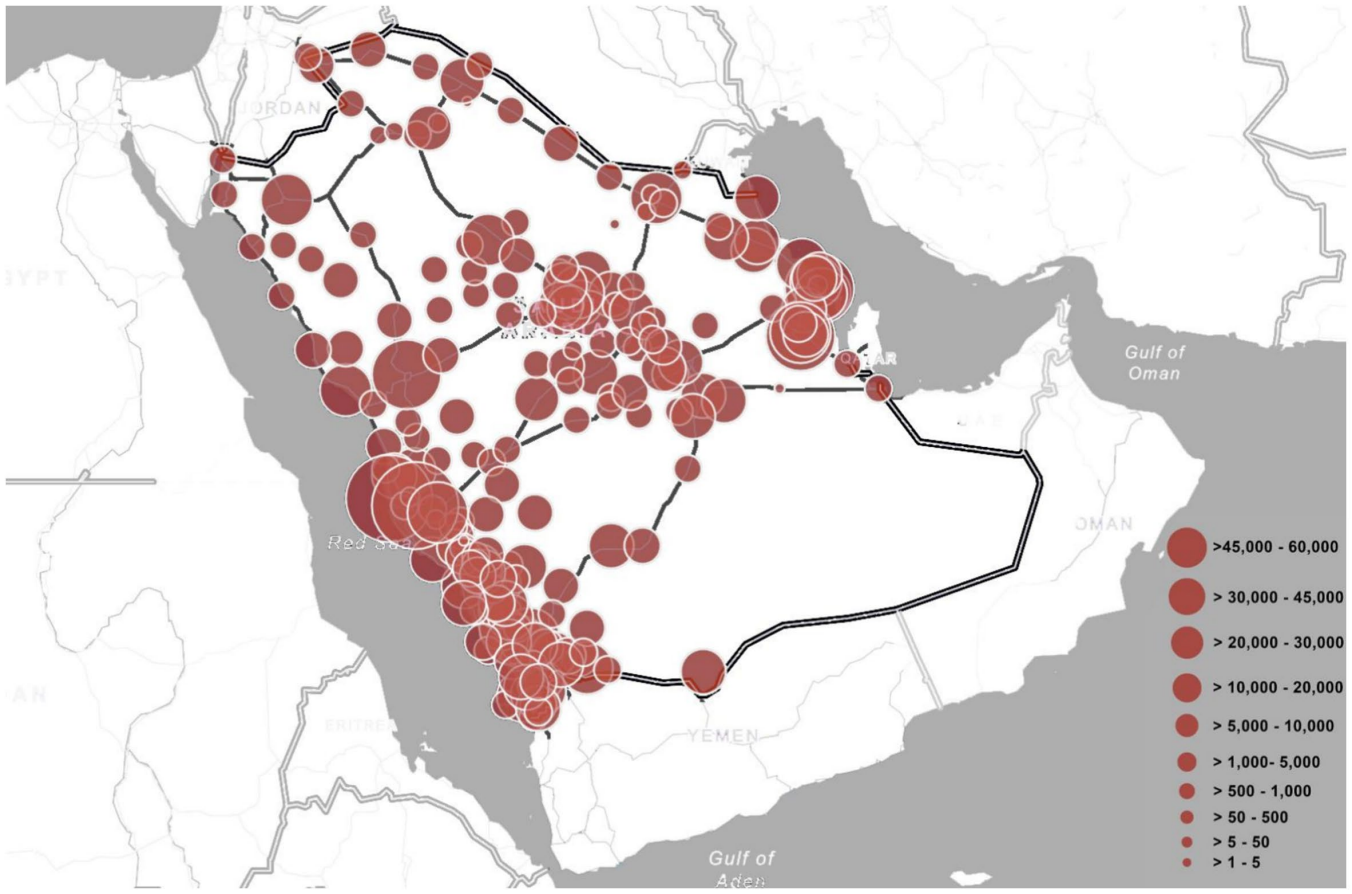
Cumulative number of cases in Saudi Arabia. The Saudi Ministry of Health (n.d.). COVID-19 dashboard. https://covid19.moh.gov.sa.

The city was the unit of analysis. Saudi Arabia has a population of around 34.8 million, spans over 2,150,000 km^2^, and is governed over 13 administrative regions and 36 major cities. There were 18 selected cities, totaling a mean population of 1,385,067 (range depends on city) and a median area of 1,994 km^2^ (Figure 1).

Principal variables of interest were:

- Exposure variables: population of the city, area of the city (km^2^), and urban fabric type (central, linear, or grid).
- Dependent variable: aggregate number of lab-confirmed COVID-19 cases up till November 2021.
- Derived variables were: Population density (population per square kilometre), Incidence per population (cases per 100 population), Incidence per area (cases per square kilometre), Cases per density group, in order to investigate distribution patterns according to urban fabric.

COVID-19 total cases data was pulled from the Saudi Ministry of Health’s official COVID-19 dashboard. Population and area figures of the respective cities were downloaded from the General Authority of Statistics. All data were visually checked for internal consistency and completeness prior to analysis.

All data available for the 18 selected cities were used; hence, no estimation of the sample size was done. Such a census-like method provided complete representation of the target cities.

Descriptive statistics (range, median, and mean) were utilized to aggregate city population, area, population density, and COVID-19 numbers. Incidence rates of COVID-19, 100 persons and per km^2^, were estimated for every city. Cities of varying types of urban fabric (central, linear, grid) were contrasted in order to investigate possible differences in distribution patterns of cases. Data analysis was done utilizing Microsoft excel and SPSS v27, and tables and figures were used to display the findings in terms of geographic and density-associated trends.

## Results

The study analyzed 18 major cities in Saudi Arabia, encompassing a total of 358,380 confirmed COVID-19 cases. The combined area of these cities was 35,890.36 km^2^, with a total population of 26,166,576.00 in 2020. Population density was quite varied, and the mean population stood at 4,948 people per square km. Across all cities, the average number of cases per square kilometer was 42. Cases per 100 population and cases per density had the respective means of 1.44% and 21, respectively. Population growth within the years 2004–2020 had an average of 1.13% among the sampled cities.

**Figure 2:**
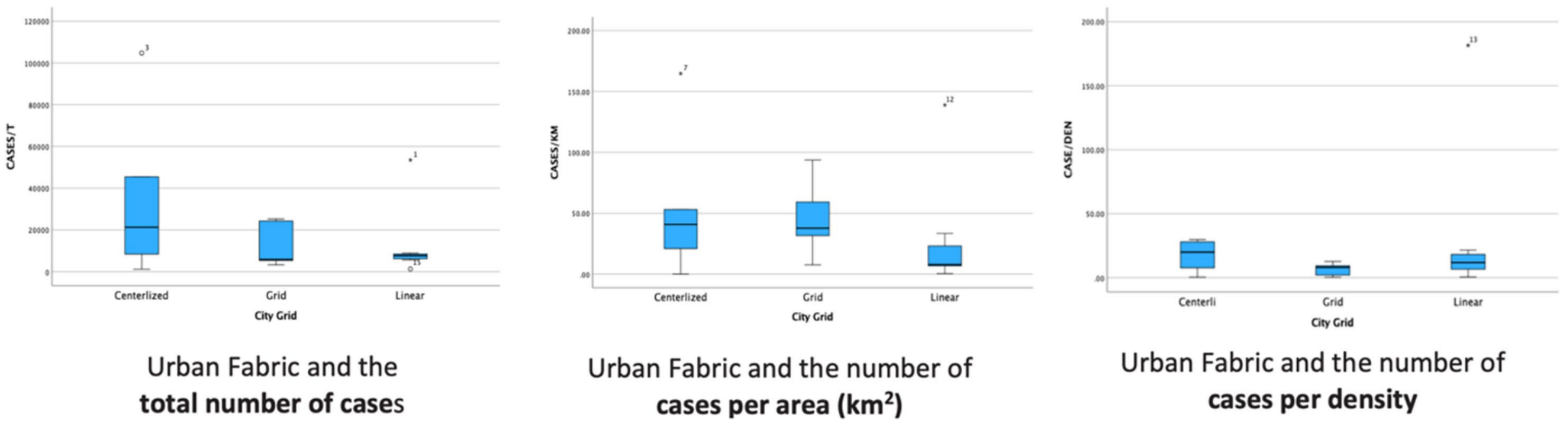
Association of urban fabric with COVID-19 cases: comparison of total cases, cases per area (km^2^), and cases per population density.

The cities were divided according to urban fabric into three groups: linear (7 cities), centralized (6 cities), and grid-patterned (5 cities). Centralized cities showed the most overall cases (Table 1), roughly thrice those of linear cities. Correspondingly, centralized cities showed the most cases per km^2^ (40.83) and cases per density (19.92 per km^2^ per 100 population) and, conversely, linear cities tended to show the fewest cases per km^2^ (7.85 cases per km^2^). Grid-patterned cities showed intermediate cases per km^2^ (37.74) and the lowest cases per density (8.08 per km^2^ per 100 population) (Figure 2).

**Table 1:** Urban fabric and number of cases.

Variables	Urban fabric category median (IQR)			Kruskal-Wallis H Test (*p*-value)
	Linear *n* = 7	Centralized *n* = 6	Grid *n* = 5	
Total cases	7,700 (3,347)	21,300 (53,660)	5,900 (20,420)	1.992 (0.369)
Cases per area (km^2^)	7.85 (27.49)	40.83 (65.16)	37.74 (56.88)	2.516 (0.284)
Cases per population	1.17 (0.39)	1.19 (1.57)	1.39 (0.90)	0.706 (0.702)
Cases per density	11.84 (19.84)	19.92 (22.53)	8.08 (9.81)	2.495 (0.287)

Case comparisons of total cases, cases per area, cases per population, and cases per density between types of urban fabric were not statistically significant (Table 1). Thus, while spatial concentration of cases relies on urban form, rates of infection in proportion to overall population size remain unaffected at a significant level by the shape of the city.

The total number of cases was assessed against the different measures in the study, i.e., cases per population density, cases per area, cases per population, and total population size (Table 2). A strong and statistically significant correlation was observed between the total number of COVID-19 cases and the totla city population size (*r* = 0.942, *p* < 0.001), indicating that cities with larger populations experienced substantially higher numbers of positive cases (Figure 2). Which in turn leads to a significant correlation between the total number of cases and the number of cases per population (*r* = 0.506, *p* = 0.032). No correlation was found between the total number of cases and cases per density (*r* = 0.410, *p* = 0.091) or cases per area (km^2^) (*r* = 0.288, *p* = 0.247).

**Table 2:** Pearson correlation of COVID-19 cases with population metrics and density.

Population and spatial metrics	Total cases Pearson correlation (*p*-value)
Cases per density	0.410 (0.091)
Cases per population	**0.506 (0.032)**
Cases per area (Km^2^)	0.288 (0.247)
Population in 2020	0.942^*^ **(<0.001)**

*Indicate strong linear correlation. Bold indicate significant associations.

## Discussion

This paper points up the intricate interrelationship between city attributes and COVID-19 transmission among Saudi Arabian cities. Similar to previous work, larger-population cities were found to have a higher risk of infection, lending support to the suggestion that population is among the most important disease-spreading catalysts of large cities (20). By virtue of their larger population sizes, naturally, there will be more potential carriers of viral transmission, and this explains the value of increasing public health measures according to the size of the city.

While population density at very high levels was linked with enhanced transmission in some global analyses (21–24), results of this study indicate the relationship might not be direct. Neighborhood connectedness and urban shape would then seem critical, and plans of central cities might promote denser human contact and larger movements, spreading transmission through the air (3). Linear or less connected sorts of cities might restrict frequent associations and slow transmission of viruses, demonstrating city planning’s ability of altering epidemiological outcomes outside of population density.

In addition to structural characteristics, pedestrian flow, use of public transit, and accessibility of concentrated central hotspots of intensive use probably shape transmission at the neighborhood scale. Such variables indicate the interplay among human behavior, environment, and city form in the determination of infection intensity. By example, hotspots of footfall or concentrated public spaces tend to speed up transmission even among comparatively dense cities, and thus infection risk cannot be accounted for by population size alone. The findings indicate the merits of taking both demographic and urban planning into account in public health interventions. Even though population is the overriding determinant, connectivity, behavioral travel, and environmental situation may limit risk, and thus interventions will need to be locally tailored based on city-level connectivity and other attributes of the urban environment. Such an approach will work best for centralized or highly connected cities, for which localized interventions, such as crowd control, restriction of travel, and localized screening, may work best in reducing transmission.

Last, the paper sheds light on large-scale pandemic preparedness. Comprehending how population dynamics and urban form work together to determine infection risk can be used to inform resilient city design, minimizing airborne transmission of disease. Future work would be valuable in examining the interplay of form and behavioral and policy variables, such as social distancing and mask use, and in deriving comprehensive models of urban epidemic disease dynamics.

## Conclusion

COVID-19, and past pandemics like the 1918 Spanish flu and 2014 Ebola outbreak, sheds light on the large-scale effect of communicable diseases on cities and the intense need for flexible city planning and public health policies (25). We studied the role of urban form in the transmission of COVID-19 among 17 major cities of Saudi Arabia, with equal representation of all municipal areas. Main variables studied were area of city, population, population density, total positive cases, cases per area, cases per population, and cases per density. Cities were divided in accordance with their city fabric into centralized, linear, and grid based.

We find there is a statistically significant relationship between population sizes of cities and total COVID-19 infections, and there is good correlation between total infections and cases/density, respectively. Population density, however, was not strongly individually correlated, though. Urban shape was also a factor in transmission dynamics and centralized (radial) cityscapes had higher infection rates than linear or grid-pattern cities. We find these findings indicate population size continues to control spreading of disease, but structure and interconnectivity of cities control regional transmission risk most especially in highly connected metropolises.

In public health and urban planning, these findings have important consequences. Policymakers and urban planners would be interested in considering the shape of the city and connectivity in designing strategies for containing the transmission of airborne communicable illness, optimizing crowd control, transit, and public space usage. Core and highly connected areas may require special measures in hopes of containing risk of transmission, particularly for future pandemics or epidemics. Future work must expand the number of cities analyzed, incorporate socio-economic variables such as income and travel trends, and examine additional environmental and behavioral transmission correlates. Combing urban form, demographic characteristics, and socio-economic variables, future research will be in a position to bring a more comprehensive framework for informing pandemic preparedness and for supporting development of resilient cities in Saudi Arabia and globally.

## Data Availability

The original contributions presented in the study are included in the article/supplementary material, further inquiries can be directed to the corresponding author.
